# 1-[(2-Chloro-7,8-dimethyl­quinolin-3-yl)meth­yl]pyridin-2(1*H*)-one

**DOI:** 10.1107/S1600536810011505

**Published:** 2010-03-31

**Authors:** F. Nawaz Khan, S. Mohana Roopan, Venkatesha R. Hathwar, Mehmet Akkurt

**Affiliations:** a Organic and Medicinal Chemistry Research Laboratory, Organic Chemistry Division, School of Advanced Sciences, VIT University, Vellore 632 014, Tamil Nadu, India; b Solid State and Structural Chemistry Unit, Indian Institute of Science, Bangalore 560 012, Karnataka, India; c Department of Physics, Faculty of Arts and Sciences, Erciyes University, 38039 Kayseri, Turkey

## Abstract

In the title compound, C_17_H_15_ClN_2_O, the quinoline ring system is nearly planar, with a maximum deviation from the mean plane of 0.074 (2) Å, and makes a dihedral angle of 81.03 (7)° with the pyridone ring. The crystal packing is stabilized by π–π stacking inter­actions between the pyridone and benzene rings of the quinoline ring system [centroid–centroid distance = 3.6754 (10) Å]. Furthermore, weak inter­molecular C—H⋯O hydrogen bonding links mol­ecules into supra­molecular chains along [001].

## Related literature

For 2-pyridone analogues, see: Arman *et al.* (2009 ▶); Clegg & Nichol (2004 ▶); Nichol & Clegg (2005 ▶). For alkaloid analogues of natural or synthetic anti­cancer agents, see: Roopan & Khan (2009 ▶). For *N*-alkyl­ation in organic synthesis, see: Roopan *et al.* (2010 ▶).
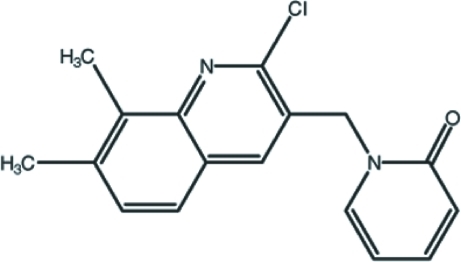


## Experimental

### 

#### Crystal data

C_17_H_15_ClN_2_O
*M* *_r_* = 298.76
Monoclinic, 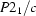

*a* = 7.07034 (17) Å
*b* = 15.4729 (4) Å
*c* = 13.1704 (3) Å
β = 96.342 (2)°
*V* = 1432.01 (6) Å^3^
*Z* = 4
Mo *K*α radiation
μ = 0.27 mm^−1^
*T* = 295 K
0.24 × 0.15 × 0.12 mm

#### Data collection

Oxford Xcalibur Eos (Nova) CCD detector diffractometer
Absorption correction: multi-scan (*CrysAlis PRO RED*; Oxford Diffraction, 2009 ▶) *T*_min_ = 0.953, *T*_max_ = 0.968
15150 measured reflections
2810 independent reflections
2008 reflections with *I* > 2σ(*I*)
*R*_int_ = 0.035

#### Refinement

*R*[*F* ^2^ > 2σ(*F* ^2^)] = 0.037
*wR*(*F* ^2^) = 0.108
*S* = 1.06
2810 reflections
192 parameters
H-atom parameters constrained
Δρ_max_ = 0.20 e Å^−3^
Δρ_min_ = −0.20 e Å^−3^

### 

Data collection: *CrysAlis PRO CCD* (Oxford Diffraction, 2009 ▶); cell refinement: *CrysAlis PRO CCD*; data reduction: *CrysAlis PRO RED*  (Oxford Diffraction, 2009 ▶); program(s) used to solve structure: *SHELXS97* (Sheldrick, 2008 ▶); program(s) used to refine structure: *SHELXL97* (Sheldrick, 2008 ▶); molecular graphics: *ORTEP-3 for Windows* (Farrugia, 1997 ▶); software used to prepare material for publication: *WinGX* (Farrugia, 1999 ▶) and *PLATON* (Spek, 2009 ▶).

## Supplementary Material

Crystal structure: contains datablocks global, I. DOI: 10.1107/S1600536810011505/xu2741sup1.cif

Structure factors: contains datablocks I. DOI: 10.1107/S1600536810011505/xu2741Isup2.hkl

Additional supplementary materials:  crystallographic information; 3D view; checkCIF report

## Figures and Tables

**Table 1:** Hydrogen-bond geometry (Å, °)

*D*—H⋯*A*	*D*—H	H⋯*A*	*D*⋯*A*	*D*—H⋯*A*
C12—H12⋯O1^i^	0.93	2.52	3.318 (2)	143

Symmetry code: (i) 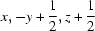.

**Fig. 1.**
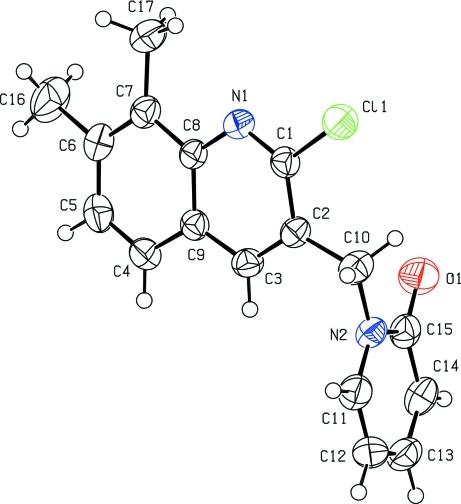
View of the title compound with the atom numbering scheme. Displacement ellipsoids for non-H atoms are drawn at the 50% probability level.

**Fig. 2.**
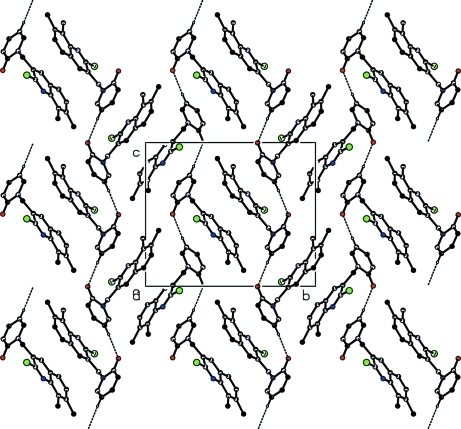
The packing and hydrogen bonding interactions of (I) viewing down the a-axis. H atoms not participating in hydrogen bonding have been omitted for clarity.

## supplementary crystallographic information

### Comment

Compounds found in nature display a wide range of diversity in terms of their structures and physical and biological properties. Several alkaloid analogues of natural or synthetic anticancer agents (Roopan & Khan, 2009) are well known, and have attracted considerable interest because of their significant activity. Particularly, five and six membered heterocyclic compounds containing one or two hetero atoms fused to a quinoline ring are found in natural products. The search for new anticancer drugs from nature continues to be a fruitful activity, as evidenced by the successes of natural products as pharmaceutical agents. Nitrogen containing heterocyclic compounds, recognized pharmacophores has received great attention in drug discovery and lead optimization. The chemistry of *N*-alkylation has recently received much attention due to their usefulness as building blocks in organic synthesis (Roopan *et al.*, 2010). On the basis of the interesting structures and biological activities exhibited by several heterocyclic systems possessing quinoline and pyridone nucleus, we have synthesized a quinoline coupled pyridone, i.e., 1-[(2-chloroquinolin-3yl)-methyl]-pyridine-2(1*H*)-one.

In the title molecule, Fig.1., the quinoline unit is nearly planar, with maximum deviations from the mean plane of -0.074 (2) Å for C2, -0.061 (2) Å for C6 and 0.059 (1) Å for N1 and 0.049 (2) Å for C4. The dihedral angle between the pyridine ring and the quinoline fused-ring system is 81.03 (7)°. Molecular conformation is stabilized by the intra molecular C—H···N and C—H···Cl interactions, forming a 5-membered ring. The crystal structure shows the presence of intermolecular π-π interactions between the pyridone (N2/C11–C15) and benzene (C4–C9) rings of the quinoline ring system, with the Cg2···Cg3(x, 1/2-y, 1/2+z) distance of 3.6754 (10) Å [Cg2 and Cg3 are the centroids of the N2/C11–C15 pyridone and C4–C9 benzene rings, respectively]. The molecular packing is further stabilized by weak intermolecular C—H···O interactions (Table1, Fig. 2), forming chains in the [001] direction.

### Experimental

To a mixed well solution of 2-pyridone (95 mg, 1 mmol, in 2 ml of DMF), KO^t^Bu (112 mg, 1 mmol in 10 ml of THF) and 2-chloro-3-(chloromethyl)-7,8-dimethylquinoline (240 mg, 1 mmol) were added and the resulting mixture was refluxed at 343 K for 1 h. After the completion of the reaction, cooled and removed the excess of solvent under reduced pressure. Crushed ice was mixed with the residue. White solid was formed, filtered and dried, purified by column chromatography using hexane and ethylacetate as the eluant. Crystals of suitable quality were grown by solvent evaporation from a solution of the compound in chloroform.

### Refinement

All H atoms were positioned geometrically and were treated as riding on their parent C atoms, with C—H = 0.93-0.97 Å and *U*_iso_(H) = 1.2 or 1.5*U*_eq_(C).

### Crystal data

**Table:**

C_17_H_15_ClN_2_O	*F*(000) = 624
*M**_r_* = 298.76	*D*_x_ = 1.386 Mg m^−^^3^
Monoclinic, *P*2_1_/*c*	Mo *K*α radiation, λ = 0.71073 Å
Hall symbol: -P 2ybc	Cell parameters from 1523 reflections
*a* = 7.07034 (17) Å	θ = 2.6–26.0°
*b* = 15.4729 (4) Å	µ = 0.27 mm^−^^1^
*c* = 13.1704 (3) Å	*T* = 295 K
β = 96.342 (2)°	Block, colourless
*V* = 1432.01 (6) Å^3^	0.24 × 0.15 × 0.12 mm
*Z* = 4	

### Data collection

**Table:**

Oxford Xcalibur Eos (Nova) CCD detector diffractometer	2810 independent reflections
Radiation source: Enhance (Mo) X-ray Source	2008 reflections with *I* > 2σ(*I*)
graphite	*R*_int_ = 0.035
ω scans	θ_max_ = 26.0°, θ_min_ = 2.6°
Absorption correction: multi-scan (*CrysAlis PRO* RED; Oxford Diffraction, 2009)	*h* = −8→8
*T*_min_ = 0.953, *T*_max_ = 0.968	*k* = −19→19
15150 measured reflections	*l* = −16→16

### Refinement

**Table:**

Refinement on *F*^2^	Primary atom site location: structure-invariant direct methods
Least-squares matrix: full	Secondary atom site location: difference Fourier map
*R*[*F*^2^ > 2σ(*F*^2^)] = 0.037	Hydrogen site location: inferred from neighbouring sites
*wR*(*F*^2^) = 0.108	H-atom parameters constrained
*S* = 1.06	*w* = 1/[σ^2^(*F*_o_^2^) + (0.060*P*)^2^] where *P* = (*F*_o_^2^ + 2*F*_c_^2^)/3
2810 reflections	(Δ/σ)_max_ < 0.001
192 parameters	Δρ_max_ = 0.20 e Å^−^^3^
0 restraints	Δρ_min_ = −0.20 e Å^−^^3^

### Special details

**Table:**

Geometry. Bond distances, angles etc. have been calculated using the rounded fractional coordinates. All su's are estimated from the variances of the (full) variance-covariance matrix. The cell esds are taken into account in the estimation of distances, angles and torsion angles
Refinement. Refinement on *F*^2^ for ALL reflections except those flagged by the user for potential systematic errors. Weighted *R*-factors wR and all goodnesses of fit *S* are based on *F*^2^, conventional *R*-factors *R* are based on *F*, with *F* set to zero for negative *F*^2^. The observed criterion of *F*^2^ > σ(*F*^2^) is used only for calculating -*R*-factor-obs etc. and is not relevant to the choice of reflections for refinement. *R*-factors based on *F*^2^ are statistically about twice as large as those based on *F*, and *R*-factors based on ALL data will be even larger.

### Fractional atomic coordinates and isotropic or equivalent isotropic displacement parameters (Å^2^)

**Table:**

	*x*	*y*	*z*	*U*_iso_*/*U*_eq_	
Cl1	1.38101 (6)	0.30106 (3)	0.46949 (4)	0.0555 (2)	
O1	0.8143 (2)	0.15968 (9)	0.50257 (10)	0.0645 (5)	
N1	1.14556 (19)	0.39838 (9)	0.35696 (10)	0.0395 (5)	
N2	0.8843 (2)	0.24988 (9)	0.63744 (10)	0.0401 (5)	
C1	1.1608 (2)	0.35195 (10)	0.43917 (13)	0.0376 (5)	
C2	1.0220 (2)	0.33920 (11)	0.50689 (12)	0.0379 (5)	
C3	0.8596 (2)	0.38656 (11)	0.48572 (12)	0.0388 (6)	
C4	0.6662 (2)	0.48857 (11)	0.37163 (14)	0.0448 (6)	
C5	0.6463 (3)	0.53389 (12)	0.28258 (14)	0.0504 (7)	
C6	0.7855 (3)	0.53271 (11)	0.21389 (14)	0.0465 (6)	
C7	0.9535 (2)	0.48792 (11)	0.23822 (13)	0.0406 (6)	
C8	0.9779 (2)	0.44201 (10)	0.33237 (12)	0.0347 (5)	
C9	0.8327 (2)	0.44006 (10)	0.39814 (12)	0.0360 (5)	
C10	1.0577 (3)	0.27637 (13)	0.59472 (14)	0.0485 (6)	
C11	0.8430 (3)	0.28316 (12)	0.72898 (14)	0.0490 (7)	
C12	0.6894 (3)	0.25858 (13)	0.77200 (14)	0.0533 (7)	
C13	0.5686 (3)	0.19603 (12)	0.72202 (15)	0.0525 (7)	
C14	0.6077 (3)	0.16224 (12)	0.63285 (14)	0.0490 (6)	
C15	0.7703 (3)	0.18774 (11)	0.58469 (13)	0.0435 (6)	
C16	0.7440 (3)	0.57702 (15)	0.11113 (15)	0.0744 (9)	
C17	1.1065 (3)	0.48403 (13)	0.16669 (14)	0.0544 (7)	
H3	0.76520	0.38360	0.52950	0.0470*	
H4	0.57050	0.48960	0.41470	0.0540*	
H5	0.53700	0.56680	0.26650	0.0600*	
H10A	1.12000	0.22540	0.57130	0.0580*	
H10B	1.14370	0.30290	0.64830	0.0580*	
H11	0.92440	0.32400	0.76200	0.0590*	
H12	0.66280	0.28230	0.83370	0.0640*	
H13	0.46110	0.17800	0.75090	0.0630*	
H14	0.52600	0.12080	0.60130	0.0590*	
H16A	0.84350	0.61760	0.10210	0.1120*	
H16B	0.62460	0.60700	0.10850	0.1120*	
H16C	0.73740	0.53460	0.05770	0.1120*	
H17A	1.06670	0.44600	0.11080	0.0820*	
H17B	1.22240	0.46280	0.20300	0.0820*	
H17C	1.12740	0.54080	0.14090	0.0820*	

### Atomic displacement parameters (Å^2^)

**Table:**

	*U*^11^	*U*^22^	*U*^33^	*U*^12^	*U*^13^	*U*^23^
Cl1	0.0391 (3)	0.0611 (4)	0.0671 (4)	0.0103 (2)	0.0101 (2)	0.0147 (2)
O1	0.0904 (11)	0.0639 (9)	0.0404 (7)	−0.0009 (8)	0.0126 (7)	−0.0064 (6)
N1	0.0385 (8)	0.0395 (8)	0.0416 (8)	−0.0019 (7)	0.0091 (6)	0.0031 (7)
N2	0.0456 (9)	0.0409 (8)	0.0341 (8)	−0.0025 (7)	0.0055 (7)	0.0053 (7)
C1	0.0338 (9)	0.0344 (9)	0.0446 (10)	0.0002 (7)	0.0046 (7)	0.0005 (8)
C2	0.0381 (9)	0.0360 (9)	0.0396 (9)	−0.0050 (8)	0.0047 (7)	0.0015 (7)
C3	0.0368 (10)	0.0403 (10)	0.0403 (9)	−0.0030 (8)	0.0090 (7)	−0.0005 (8)
C4	0.0429 (10)	0.0416 (10)	0.0512 (11)	0.0045 (8)	0.0106 (8)	−0.0010 (9)
C5	0.0489 (11)	0.0394 (11)	0.0617 (12)	0.0082 (9)	0.0012 (9)	0.0035 (9)
C6	0.0527 (11)	0.0379 (10)	0.0475 (11)	−0.0025 (9)	−0.0001 (9)	0.0069 (8)
C7	0.0452 (10)	0.0355 (10)	0.0409 (10)	−0.0057 (8)	0.0045 (8)	0.0004 (8)
C8	0.0357 (9)	0.0305 (9)	0.0383 (9)	−0.0026 (7)	0.0053 (7)	0.0007 (7)
C9	0.0357 (9)	0.0310 (9)	0.0413 (9)	−0.0027 (7)	0.0043 (7)	−0.0024 (7)
C10	0.0410 (10)	0.0559 (12)	0.0483 (11)	−0.0010 (9)	0.0041 (8)	0.0133 (9)
C11	0.0636 (13)	0.0443 (11)	0.0388 (10)	−0.0033 (10)	0.0042 (9)	−0.0013 (8)
C12	0.0699 (14)	0.0542 (12)	0.0382 (10)	0.0064 (10)	0.0166 (10)	0.0012 (9)
C13	0.0506 (12)	0.0581 (13)	0.0502 (12)	0.0049 (10)	0.0116 (9)	0.0191 (10)
C14	0.0526 (12)	0.0477 (11)	0.0445 (10)	−0.0078 (9)	−0.0049 (9)	0.0098 (9)
C15	0.0538 (11)	0.0414 (10)	0.0342 (9)	0.0028 (9)	0.0000 (8)	0.0044 (8)
C16	0.0791 (16)	0.0780 (16)	0.0635 (14)	0.0081 (13)	−0.0036 (12)	0.0300 (12)
C17	0.0606 (13)	0.0582 (12)	0.0463 (11)	−0.0009 (10)	0.0139 (10)	0.0102 (9)

### Geometric parameters (Å, °)

**Table:**

Cl1—C1	1.7511 (15)	C11—C12	1.335 (3)
O1—C15	1.237 (2)	C12—C13	1.405 (3)
N1—C1	1.294 (2)	C13—C14	1.342 (3)
N1—C8	1.372 (2)	C14—C15	1.429 (3)
N2—C10	1.463 (3)	C3—H3	0.9300
N2—C11	1.372 (2)	C4—H4	0.9300
N2—C15	1.390 (2)	C5—H5	0.9300
C1—C2	1.411 (2)	C10—H10A	0.9700
C2—C3	1.365 (2)	C10—H10B	0.9700
C2—C10	1.511 (3)	C11—H11	0.9300
C3—C9	1.415 (2)	C12—H12	0.9300
C4—C5	1.360 (3)	C13—H13	0.9300
C4—C9	1.407 (2)	C14—H14	0.9300
C5—C6	1.409 (3)	C16—H16A	0.9600
C6—C7	1.382 (3)	C16—H16B	0.9600
C6—C16	1.517 (3)	C16—H16C	0.9600
C7—C8	1.423 (2)	C17—H17A	0.9600
C7—C17	1.512 (3)	C17—H17B	0.9600
C8—C9	1.415 (2)	C17—H17C	0.9600
			
Cl1···C3^i^	3.6175 (15)	C11···H3	3.0500
Cl1···C14^i^	3.324 (2)	C15···H17A^v^	2.9400
Cl1···C15^i^	3.468 (2)	C16···H17A	3.0500
Cl1···H3^i^	3.0300	C16···H17C	2.7500
Cl1···H10A	2.6700	C17···H16A	2.8500
Cl1···H10B	3.0400	C17···H16C	2.9400
Cl1···H12^ii^	3.1000	H3···Cl1^vi^	3.0300
Cl1···H13^ii^	3.0100	H3···N2	2.6000
O1···C2	3.140 (2)	H3···C11	3.0500
O1···C12^iii^	3.318 (2)	H3···H4	2.5300
O1···H10A	2.4700	H4···H3	2.5300
O1···H17C^iv^	2.7000	H4···H4^viii^	2.5800
O1···H12^iii^	2.5200	H5···H16B	2.3200
O1···H17A^v^	2.7100	H10A···Cl1	2.6700
N1···H17B	2.3800	H10A···O1	2.4700
N2···H3	2.6000	H10B···Cl1	3.0400
C2···O1	3.140 (2)	H10B···H11	2.2900
C3···Cl1^vi^	3.6175 (15)	H10B···C5^vii^	3.0200
C3···C11	3.595 (2)	H11···H10B	2.2900
C3···C15	3.427 (2)	H11···C6^vii^	3.0100
C4···C13^iii^	3.495 (3)	H11···C7^vii^	3.0400
C6···C14^iii^	3.394 (3)	H11···H16A^vii^	2.4600
C7···C14^iii^	3.543 (3)	H11···H17C^vii^	2.5000
C7···C15^iii^	3.546 (2)	H12···Cl1^ix^	3.1000
C9···C13^iii^	3.513 (2)	H12···O1^v^	2.5200
C9···C12^iii^	3.586 (3)	H13···Cl1^ix^	3.0100
C11···C3	3.595 (2)	H16A···C17	2.8500
C12···O1^v^	3.318 (2)	H16A···H17C	2.3400
C12···C9^v^	3.586 (3)	H16A···H11^vii^	2.4600
C13···C9^v^	3.513 (2)	H16B···H5	2.3200
C13···C4^v^	3.495 (3)	H16C···C17	2.9400
C14···C6^v^	3.394 (3)	H17A···C16	3.0500
C14···C7^v^	3.543 (3)	H17A···O1^iii^	2.7100
C14···Cl1^vi^	3.324 (2)	H17A···C15^iii^	2.9400
C15···C3	3.427 (2)	H17B···N1	2.3800
C15···C7^v^	3.546 (2)	H17C···C16	2.7500
C15···Cl1^vi^	3.468 (2)	H17C···H16A	2.3400
C5···H10B^vii^	3.0200	H17C···O1^x^	2.7000
C6···H11^vii^	3.0100	H17C···H11^vii^	2.5000
C7···H11^vii^	3.0400		
			
C1—N1—C8	117.44 (13)	N2—C15—C14	114.82 (15)
C10—N2—C11	120.11 (15)	C2—C3—H3	119.00
C10—N2—C15	117.76 (14)	C9—C3—H3	120.00
C11—N2—C15	122.06 (15)	C5—C4—H4	120.00
Cl1—C1—N1	115.48 (12)	C9—C4—H4	120.00
Cl1—C1—C2	117.13 (12)	C4—C5—H5	119.00
N1—C1—C2	127.38 (14)	C6—C5—H5	119.00
C1—C2—C3	115.09 (15)	N2—C10—H10A	109.00
C1—C2—C10	120.33 (14)	N2—C10—H10B	109.00
C3—C2—C10	124.58 (15)	C2—C10—H10A	109.00
C2—C3—C9	120.99 (14)	C2—C10—H10B	109.00
C5—C4—C9	119.53 (16)	H10A—C10—H10B	108.00
C4—C5—C6	122.27 (18)	N2—C11—H11	119.00
C5—C6—C7	120.08 (17)	C12—C11—H11	119.00
C5—C6—C16	119.16 (18)	C11—C12—H12	121.00
C7—C6—C16	120.68 (17)	C13—C12—H12	121.00
C6—C7—C8	118.09 (15)	C12—C13—H13	120.00
C6—C7—C17	122.09 (16)	C14—C13—H13	120.00
C8—C7—C17	119.79 (14)	C13—C14—H14	119.00
N1—C8—C7	118.03 (13)	C15—C14—H14	119.00
N1—C8—C9	120.77 (14)	C6—C16—H16A	110.00
C7—C8—C9	121.19 (13)	C6—C16—H16B	109.00
C3—C9—C4	123.20 (14)	C6—C16—H16C	110.00
C3—C9—C8	118.04 (13)	H16A—C16—H16B	109.00
C4—C9—C8	118.71 (14)	H16A—C16—H16C	109.00
N2—C10—C2	113.49 (15)	H16B—C16—H16C	109.00
N2—C11—C12	121.90 (18)	C7—C17—H17A	109.00
C11—C12—C13	118.58 (18)	C7—C17—H17B	109.00
C12—C13—C14	120.33 (19)	C7—C17—H17C	109.00
C13—C14—C15	122.32 (18)	H17A—C17—H17B	110.00
O1—C15—N2	119.46 (18)	H17A—C17—H17C	109.00
O1—C15—C14	125.72 (17)	H17B—C17—H17C	109.00
			
C8—N1—C1—Cl1	−177.63 (11)	C9—C4—C5—C6	1.4 (3)
C8—N1—C1—C2	1.3 (2)	C5—C4—C9—C8	1.9 (2)
C1—N1—C8—C7	−175.25 (15)	C5—C4—C9—C3	−175.64 (16)
C1—N1—C8—C9	3.7 (2)	C4—C5—C6—C7	−3.3 (3)
C10—N2—C11—C12	−177.86 (18)	C4—C5—C6—C16	173.50 (18)
C11—N2—C15—C14	0.5 (2)	C16—C6—C7—C17	2.7 (3)
C10—N2—C15—O1	−2.8 (2)	C5—C6—C7—C8	1.6 (3)
C15—N2—C11—C12	−0.9 (3)	C5—C6—C7—C17	179.44 (17)
C11—N2—C15—O1	−179.78 (17)	C16—C6—C7—C8	−175.12 (16)
C10—N2—C15—C14	177.47 (16)	C6—C7—C8—C9	1.7 (2)
C11—N2—C10—C2	−104.43 (18)	C17—C7—C8—N1	2.8 (2)
C15—N2—C10—C2	78.5 (2)	C6—C7—C8—N1	−179.28 (15)
N1—C1—C2—C3	−5.0 (3)	C17—C7—C8—C9	−176.16 (15)
N1—C1—C2—C10	174.62 (16)	C7—C8—C9—C4	−3.5 (2)
Cl1—C1—C2—C10	−6.5 (2)	C7—C8—C9—C3	174.16 (15)
Cl1—C1—C2—C3	173.96 (12)	N1—C8—C9—C4	177.54 (15)
C1—C2—C3—C9	3.5 (2)	N1—C8—C9—C3	−4.8 (2)
C1—C2—C10—N2	−161.79 (15)	N2—C11—C12—C13	0.7 (3)
C3—C2—C10—N2	17.8 (2)	C11—C12—C13—C14	−0.2 (3)
C10—C2—C3—C9	−176.05 (16)	C12—C13—C14—C15	−0.3 (3)
C2—C3—C9—C4	178.47 (16)	C13—C14—C15—O1	−179.63 (19)
C2—C3—C9—C8	0.9 (2)	C13—C14—C15—N2	0.1 (3)

Symmetry codes: (i) *x*+1, *y*, *z*; (ii) *x*+1, −*y*+1/2, *z*−1/2; (iii) *x*, −*y*+1/2, *z*−1/2; (iv) −*x*+2, *y*−1/2, −*z*+1/2; (v) *x*, −*y*+1/2, *z*+1/2; (vi) *x*−1, *y*, *z*; (vii) −*x*+2, −*y*+1, −*z*+1; (viii) −*x*+1, −*y*+1, −*z*+1; (ix) *x*−1, −*y*+1/2, *z*+1/2; (x) −*x*+2, *y*+1/2, −*z*+1/2.

### Hydrogen-bond geometry (Å, °)

**Table:**

*D*—H···*A*	*D*—H	H···*A*	*D*···*A*	*D*—H···*A*
C3—H3···N2	0.93	2.60	2.902 (2)	100
C10—H10A···Cl1	0.97	2.67	2.987 (2)	100
C12—H12···O1^v^	0.93	2.52	3.318 (2)	143
C17—H17B···N1	0.96	2.38	2.821 (2)	108

Symmetry codes: (v) *x*, −*y*+1/2, *z*+1/2.
